# Seasonal Spatio-Temporal Model Improves Refined Stock Assessment and Management of Japanese Sardine (*Sardinops melanostictus*) in the Northwest Pacific Ocean

**DOI:** 10.3390/ani14233434

**Published:** 2024-11-27

**Authors:** Yongchuang Shi, Qingpeng Han, Shengmao Zhang, Shenglong Yang, Tianfei Cheng, Wei Fan, Guoqing Zhao, Haibin Han, Heng Zhang

**Affiliations:** 1 East China Sea Fisheries Research Institute, Chinese Academy of Fishery Sciences, Shanghai 200090, China; syc13052326091@163.com (Y.S.); zhangsm@ecsf.ac.cn (S.Z.); ysl6782195@126.com (S.Y.); chengtianfeinuist@126.com (T.C.); fanw@ecsf.ac.cn (W.F.); zgq617717@163.com (G.Z.); 2 Key Laboratory of Fisheries Remote Sensing Ministry of Agriculture and Rural Affairs, East China Sea Fisheries Research Institute, Chinese Academy of Fishery Science, Shanghai 200090, China; 3 Yellow Sea Fisheries Research Institute, Chinese Academy of Fishery Sciences, Qingdao 266071, China; hanqp@ysfri.ac.cn

**Keywords:** *Sardinops melanostictus*, seasonal spatio-temporal model, seasonal migration, stock assessment, data-limited method, ecosystem management

## Abstract

Accurate estimation of fish stock abundance and exploitability is critical for effective fishery management; however, fishery-dependent data are often affected by temporal and spatial heterogeneities due to the seasonal migration of fish, posing challenges for refined stock management. Previous studies have largely ignored these spatio-temporal dynamics, assuming static populations. This study develops a seasonal spatio-temporal model for Japanese sardine (*Sardinops melanostictus*) in the Northwest Pacific Ocean (NPO), using fishery-dependent data from 2014 to 2022. Seasonal standardized abundance indices (spring, summer, autumn, and an overall average) were generated and integrated with the abundance maximum sustainable yield (AMSY) method for stock assessment. The performance of the AMSY method using a spatio-temporal index was compared with the conventional model-based index, showing the superiority of the spatio-temporal approach. Results indicate a gradual increase in sardine abundance, with a significant shift in the center of gravity toward the northeast. The stock is in a sustainable state with a 94.8–99% probability of recovery. Although the stock is recovering, careful management is advised to prevent future declines. This framework offers a novel approach for assessing distant water and coastal fishery resources.

## Simple Summary

This study presents a refined approach for assessing Japanese sardine (*Sardinops melanostictus*) stock in the Northwest Pacific Ocean (NPO), addressing seasonal and spatial variations in abundance. We developed a seasonal spatio-temporal model that accounts for migration patterns to generate seasonal abundance indices, which were applied in the AMSY stock assessment method. The results indicate a steady increase in sardine abundance, with a significant northeast shift in the population center of gravity, confirming a sustainable stock status with a 94.8–99% recovery probability. The study underscores the benefits of spatio-temporal indices over conventional models for precise stock assessments and highlights the need for cautious management to ensure long-term stock health, given the historical fluctuations in Japanese sardine abundance. This framework offers an innovative method for managing distant-water fisheries and supporting offshore fishery research.

## 1. Introduction

Fishery stock assessment is a crucial foundation for fishery managers to formulate scientific management strategies [1]. In the context of the marine law [2], which calls for the management of exploited fish stocks, gaining accurate insights, and monitoring the stock status is of vital significance for the sustainable development of marine fisheries and the maintenance of ecosystem health [3]. However, current stock assessments for many species still suffer from inadequate data availability and poor understanding of spatio-temporal population dynamics. This has led to persistent challenges in making precise and timely management decisions. Most global fishery resource assessments, which rely on conventional integrated models, face substantial difficulties due to the lack of data such as age structure, long-term catch or resource abundance, and precise life history parameters [4,5]. To meet the pressing demand for fishery resource assessment and management, numerous data-limited methods have been developed for estimating the population stock status of data-limited fisheries [6,7].

Currently, data-limited methods have been extensively applied in the stock status assessment and management of data-poor fisheries, which are mainly divided into catch-based models and length-based models [8]. The catch-based methods can apply statistical catch data and auxiliary information (e.g., intrinsic growth rate (*r*) and natural mortality coefficients) to assess the maximum sustainable yield (MSY) and associated biological reference points (e.g., relative biomass (*B*/*B*_MSY_) and relative fishing mortality (*F*/*F*_MSY_)) of the target stock [9,10]. For example, Zhang et al. [11] estimated the MSY values for three fisheries in the East China Sea using the Catch-Maximum Sustainable Yield (Catch-MSY) model and proposed recommendations for their population management. Arnold et al. [12] utilized the depletion-corrected average catch (DCAC) and depletion-based stock reduction analysis (DB-SRA) to assess the resource status of the canary rockfish (*Sebastes pinniger*). The length-based methods can be employed to evaluate growth, mortality parameters, and exploited status of fish populations using body length frequency data [13]. For instance, Baibbat et al. [14] used the length-based spawning potential ratio (LBSPR) model to assess the Atlantic bonito (*Sarda sarda*) population along the Moroccan Atlantic coast, revealing overfishing, as a substantial portion of the fish is caught both before reaching maximum growth rates and after reaching MSY. Liang et al. [15] utilized the length-based Bayesian (LBB) model to assess 14 species of fish and invertebrates in China’s coastal waters based on length frequency data. The abundance maximum sustainable yield method (AMSY) developed by Froese et al. [16] only requires the resource abundance index of a certain time series, the prior information of *r*, and the relative population biomass (*B*_t_/*K*) in a specific year to estimate the stock status of the target species more accurately; it is also an effective tool for estimating the status of data-limited fisheries. Zhou et al. [17] demonstrated that data-limited models can provide reasonably accurate parameter estimates compared to comprehensive resource assessments; therefore, data-limited methods are gaining increasing attention from global fisheries resource researchers, making it one of the hot directions of fishery resources research.

As widely acknowledged, fish populations display intricate spatio-temporal structures resulting from factors such as migration and climate change [18,19]. However, many studies have failed to capture these complexities, particularly seasonal variations that significantly impact stock assessments. Traditional assessments often treat fish populations and study regions as homogenous units, overlooking migration patterns and spatial heterogeneity [20]. This limits their effectiveness in managing highly migratory species. According to research by Lilly et al. [21] and Ames [22], it has been found that fish populations exhibit regular movements or migrations. Additionally, many marine fish species exhibit seasonal movements, migrating between different areas at various times of the year for spawning and feeding [23]. All these situations can potentially influence the standardization of abundance indices and the assessment of population status, especially for data-poor fisheries. Failure to explicitly account for the seasonal spatio-temporal variations in population dynamics can impact the accuracy of abundance indices, leading to potentially incorrect assessment of stock status. Ultimately, this can affect the development of management strategies and have significant adverse consequences for the sustainable utilization of marine fishery resources [24]; hence, utilizing seasonal spatio-temporal models that consider both seasonal and annual variations is an urgently needed solution for abundance index standardization and stock assessment.

Japanese sardine (*Sardinops melanostictus*) is a significant economic species in the Northwest Pacific Ocean (NPO), characterized by its short life cycle, rapid growth, extensive migration range, and substantial ecological value within the ecosystem [25,26,27]. As a small ocean pelagic fish, the Japanese sardine is an important source of high-quality animal protein and presents an increasingly essential dedication to food security. This fishery has a long development history in coastal countries, including China (starting in the 1990s) and Japan (starting in the 1950s), and is also an important part of the pelagic fishery in these countries [28]. According to the Food and Agriculture Organization (FAO) and the North Pacific Fisheries Commission (NPFC), Japanese sardine catches experienced a sharp decline starting in the 1990s. The catch remained at relatively low levels—below 500 thousand tons—from 1995. However, after 2010, Japanese sardine catches began to gradually recover, with total landings exceeding one million tons by 2020, a trend that has persisted into recent years. In China, the average Japanese sardine catch from 2020 to 2022 was approximately 200 thousand tons. The primary fishing method employed was lighting purse seine, with the main fishing grounds located between 147° to 153° E and 39° to 43° N. The peak fishing season typically spans from May to November [28]. Regarding the migration mentioned above, during the winter, Japanese sardines spawn near the Japanese coastal waters, subsequently migrating northeastward to the convergence of the Kuroshio and Oyashio currents in spring. In the summer, they continue their migration northeastward to feeding grounds, and as the water temperature decreases in autumn, they initiate their migration southwestward to warmer waters [29,30]. The seasonal migrations of Japanese sardines result in spatio-temporal variations in distribution, complicating the stock assessments of its fishery. If the seasonal changes in the population are not fully considered, it will have a great negative impact on the stock assessment and management. Presently, the Japanese sardine has been listed as a priority managed species by the North Pacific Fisheries Commission (NPFC), and a series of researches on resource abundance index standardization and stock assessment have been facilitated with the aim of achieving sustainable utilization of this fishery resource [31]. However, previous studies treated the NPO as a single, undifferentiated study area without considering the seasonal spatio-temporal distribution differences in Japanese sardine populations resulting from their migrations.

Model-based methods and design-based methods are currently the main methods for estimating fishery abundance indices. The working principles of these methods are different. Model-based methods use hypothetical statistical models to analyze the differences in data and then estimate them through the probability function of the response variables [32]. Design-based methods, on the other hand, usually assume that the population quantities are fixed, and the average value of each stratify is calculated by area weighting [33]. The resource abundance indices obtained by these two methods are obviously different. With the development of computer technology and statistics, model-based methods are increasingly applied to the estimation of the fishery resource abundance index [34]. Previous models in the spatio-temporal domain typically focused solely on either interannual changes [35] or seasonal changes [36]. Additionally, they did not account for the entire scope of correlations, such as ignoring relations among different years for a specified season [37], overlooking correlations among various seasons within a given year [38], or disregarding the order of seasons within the given year. For this reason, a novel method for seasonal spatio-temporal analysis that encapsulates the density of annual, seasonal, and spatial variations was provided in 2020 [39]. This model includes variations in spatial distributions in different years (interannual variations) and different seasons (seasonal variations). Through case studies and simulation tests, the excellent performance of this method in pinpointing variations in seasonal timing of fish migration and ecosystem productivity, driven by climatic factors, is demonstrated and affirmed. It is also a promising tool for the standardization of the seasonal resource abundance index and scientific stock assessment [39].

In order to address the growing need for a refined assessment of Japanese sardines in the NPO, we start by constructing a seasonal spatio-temporal model for Japanese sardines that aims to obtain precise seasonal resource abundance indices for Japanese sardine fishery. We then employ the AMSY method to evaluate the stock status of Japanese sardines across different seasons and to obtain the seasonal stock status of Japanese sardines from 2014 to 2022. The performance of stock assessment under spatio-temporal indices and conventional model-based indices is also compared. Finally, we present refined management strategy recommendations from a comprehensive perspective for fishermen, fishery managers, and fishery scientists. This study fills a significant gap by addressing seasonal dynamics, a feature often overlooked in previous stock assessments, and provides improved recommendations for management strategies that are more reflective of the biological and ecological realities of Japanese sardine fishery. Our results offer technical support for the sustainable management and population recovery of Japanese sardines, and the protection of the NPO ecosystem.

## 2. Materials and Methods

### 2.1. Study Area and Data Resource

#### 2.1.1. Study Area

**Figure 1:**
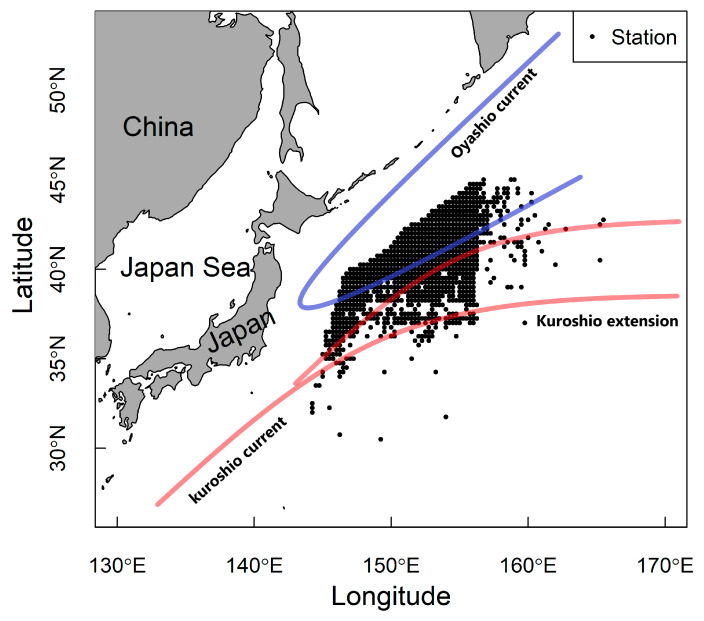
Study area. The black points represent the sampling locations for Japanese sardines. The red lines represent the Kuroshio Current and Kuroshio Extension, and the blue lines represent the Oyashio Current.

The study area is mainly distributed at the confluence of the Kuroshio warm current and the Oyashio cold current (Figure 1), which bring abundant nutrients to marine pelagic fish, making it one of the most productive areas in the world’s marine fishing industry, which is why the NPO is not only the habitat of Japanese sardines but also the main fishing area for economically important species such as Chub mackerel (*Scomber japonicas*), Pacific saury (*Cololabis saira*), and neon flying squid (*Ommastrephes bartramii*). For this study, operating locations for Japanese sardine fisheries were primarily bounded by 34°–46° N and 144° E–163° E in the NPO (Figure 1).

#### 2.1.2. Data Resource

The Technical Group for Trawl-purse Seine Fishery of Distant-water Fishery Society of China provided fisheries data for Japanese sardines from March to November 2014–2022. Because China began fishing for Japanese sardines in the NPO at a certain scale in 2014, the temporal range of the data is from 2014 to 2022. The company name, vessel number, operating location (longitude and latitude), operating time (year, month, day), yield (tons), haul, and fishing vessel length (m) were included in the fishery data information. The temporal and spatial resolutions of fishery data were month and 0.25° × 0.25°, respectively. The nominal abundance indices of Japanese sardines within a 0.25° × 0.25° grid were obtained using the following formula (the unit of abundance index was kg per vessel per day):

(1) $$CPUE=\frac{\sum Catch}{\sum Fishing\;effort}$$

where ∑Catch represents total catch within one grid and ∑Fishing effort represents total fishing days within one grid.

### 2.2. Conventional Model-Based Index

In this study, the generalized additive model (GAM) was used as a traditional model to estimate standardized CPUE:

(2) $$\log\left(CPUE\right)=Year+Month+Longitude+Latitude+Vessellength+interaction+\varepsilon$$

where ε represents the residual, which is assumed to follow a normal distribution. The term “interaction” refers to the interactive effect of spatial and temporal factors for the Japanese sardine CPUE. The full interaction model includes all possible combinations of Year, Month, Longitude, and Latitude. The interaction terms are presented in the form of “*Year×Month*”, “*Year×Longitude*”, “*Year×Latitude*”, “*Month×Longitude*”, “*Month*×*Latitude*” and “*Longitude×Latitude*” in the formula, which is used to construct the traditional CPUE standardization model. The traditional model-based index was obtained via the predicted function in the R (V4.4.0) programming environment. The R packages used for this study are “nlme” and “mgcv”.

### 2.3. Seasonal Spatio-Temporal Modeling

To achieve the goal of refined assessment and management of Japanese sardines, the fishery data were divided into spring (March to May), summer (June to August), and autumn (September to November) according to the characteristics of Japanese sardine fishery, and the seasonal spatio-temporal model developed by Thorson et al. [39] was used to standardize three seasonal resource abundance indices of Japanese sardines in the NPO, which were then used as model inputs to carry out the seasonal stock assessment. In this seasonal spatio-temporal model, a Poisson-link delta model was used to undertake the *B* to represent the likelihood of response variable $b_{i}$ for each sample *i*, $Pr\left(B=b_{i}\right)$. This method considers two main components: probability $p_{i}$ that sample *i* encounters Japanese sardine (i.e., $Pr\left(B>0\right)$) and the expected measurement $c_{i}$ when the Japanese sardine was encountered (i.e., $Pr\left(B|B\right)>0$) [39]:

(3) PrB=bi=1−pipi×gBci,σm2 if B=0if B>0

where $b_{i}$ is the abundance in the *i*th sample, $p_{i}$ is the encounter probability, $c_{i}$ represents the positive catch rate, and $\sigma_{m}^{2}$ is variance in the *m*th group. The Poisson-link delta model calculates $p_{i}$ and $c_{i}$ in two steps, first estimating $log\left(n_{i}\right)$ and $log\left(w_{i}\right)$ for each sample *I* by log-linked function, and then calculating $p_{i}$ and $c_{i}$ via $n_{i}$ and $w_{i}$.

The formulas for $n_{i}$ and $w_{i}$ are provided below:

(4) $$log\left(n_{i}\right)=\underset{\begin{array}{c} Year-Season\\ intercept \end{array}}{\underbrace{\beta_{n}^{*}\left(t_{i}\right)}}+\underset{\begin{array}{c} Spetial\;main\\ effect \end{array}}{\underbrace{\omega_{n}^{*}\left(s_{i}\right)}}+\underset{\begin{array}{c} Season\;spatial\\ effect \end{array}}{\underbrace{\xi_{nu}^{*}\left({s_{i},u}_{i}\right)}}+\underset{\begin{array}{c} Year\;spatial\\ effect \end{array}}{\underbrace{\xi_{ny}^{*}\left(s_{i},y_{i}\right)}}+\underset{\begin{array}{c} Year-Season\\ spatial\;effect \end{array}}{\underbrace{\varepsilon_{n}^{*}\left(s_{i},t_{i}\right)}}+\underset{Vessel\;effect}{\underbrace{r_{n}^{*}\left(V_{i}\right)}}$$

(5) $$log\left(w_{i}\right)=\underset{\begin{array}{c} Year-Season\\ intercept \end{array}}{\underbrace{\beta_{w}^{*}\left(t_{i}\right)}}+\underset{\begin{array}{c} Spetial\;main\\ effect \end{array}}{\underbrace{\omega_{w}^{*}\left(s_{i}\right)}}+\underset{\begin{array}{c} Season\;spatial\\ effect \end{array}}{\underbrace{\xi_{wu}^{*}\left({s_{i},u}_{i}\right)}}+\underset{\begin{array}{c} Year\;spatial\\ effect \end{array}}{\underbrace{\xi_{wy}^{*}\left(s_{i},y_{i}\right)}}+\underset{\begin{array}{c} Year-Season\\ spatial\;effect \end{array}}{\underbrace{\varepsilon_{w}^{*}\left(s_{i},t_{i}\right)}}+\underset{Vessel\;effect}{\underbrace{c_{w}^{*}\left(V_{i}\right)}}$$

where $n_{i}$ denotes the abundance density, $w_{i}$ represents the average weight, $s_{i}$ denotes the location of the *i*th sample, $t_{i}$ represents the year–season of the *i*th sample, $u_{i}$ represents the season of the *i*th sample, and $y_{i}$ represents the year. In the construction of the Japanese sardine seasonal model, we considered the impact of different vessel lengths on resource abundance ($r_{w}^{*}\left(V_{i}\right)$) then we calculated $p_{i}$ and $c_{i}$ using the following equations:

(6) $$p_{i}=1-exp\left(-a_{i}\times n_{i}\right)$$

(7) $$c_{i}=\frac{a_{i}\times n_{i}}{p_{i}}\times w_{i}$$

where $a_{i}$ represents the area-swept offset for sample *i*.

We used the vector autoregressive spatio-temporal (VAST) package (version 3.6.0) [40] in R software (V4.03) to construct a seasonal spatio-temporal model of Japanese sardines and estimate the model parameters. According to the characteristics of VAST, we specify all grid cells as 100 knots, which can not only improve the calculation efficiency of the model but also balance the spatial resolution (the more knots, the higher the spatial resolution). VAST computes fixed effects and concurrently approximates their marginal likelihood using the Laplace approximation method [41]. The Laplace approximation technique is executed using the R package TMB [42]. To enhance computational efficiency, the process employs automatic differentiation [43] and the SPDE approximation to spatial correlation matrices from R-INLA [44]. The convergence of the seasonal model was identified by (1) verifying that the final gradient for each fixed effect was at most 0.0001, and (2) ensuring that the Hessian matrix, which comprises the second derivatives of the negative log-likelihood, was definitely exhibiting positive values.

The Japanese sardine stock’s standardized total biomass/abundance in time combinations *t*, $D\left(t\right)$ was predicted as follows:

(8) $$\begin{array}{c} D\left(t\right)=\sum_{s=1}^{n_{s}}a\left(s\right)\times n\left(s,t\right)\times w\left(s,t\right)=\sum_{s=1}^{n_{s}}a\left(s\right)\times exp\left\{\beta_{n}^{*}\left(t\right)+\omega_{n}^{*}\left(s\right)+\xi_{nu}^{*}\left(s,u\right)+\xi_{ny}^{*}\left(s,y\right)+\varepsilon_{n}^{*}\left(s,t\right)\right\}\times \\ exp\left\{\beta_{w}^{*}\left(t\right)+\omega_{w}^{*}\left(s\right)+\xi_{wu}^{*}\left(s,u\right)+\xi_{wy}^{*}\left(s,y\right)+\varepsilon_{w}^{*}\left(s,t\right)\right\} \end{array}$$

where $n_{s}$ serves as the fine-scale predictions number and $a\left(s\right)$ represents the spatial area related to each prediction. $\beta_{n}^{*}\left(t\right)$, $\omega_{n}^{*}\left(s\right)$, $\xi_{nu}^{*}\left(s,u\right)$, $\xi_{ny}^{*}\left(s,y\right)$ and $\varepsilon_{n}^{*}\left(s,t\right)$ represent the year–season intercept, the spatial main effect, the season spatial effect, the year spatial effect, and the year–season spatial effect of $n\left(s,t\right)$, respectively. Similarly, the various variables of $w\left(s,t\right)$ correspond to these representations. The seasonal spatio-temporal model can extrapolate information from multiple surveys to obtain resource abundance estimates for areas lacking catch data. To accurately reflect the resource abundance of the main fishing grounds for Japanese sardines in the NPO, we selected the area between 32°–47° N and 145°–160° E for extrapolation to calculate the resource abundance in this region. In order to analyze the seasonal variations in distribution patterns of Japanese sardines, we calculated the longitudinal and latitudinal centers of gravity (COGs), and the non-parametric Mann–Kendall trend test was used to identify the variation degree of COG trends [45,46]. The Mann–Kendall trend test is a non-parametric statistical method used to detect monotonic trends (either increasing or decreasing) in time series data. It does not require the data to follow any specific distribution, making it particularly suitable for data that are non-normally distributed, discrete, or contain outliers. This process was run using the “Kendall” package in R software (V4.03).

### 2.4. AMSY Method for Japanese Sardines

Based on the outputs of seasonal spatio-temporal models, we facilitated the stock assessment of Japanese sardines by employing the AMSY model. According to the basic assumptions of the surplus production model, in the AMSY, the biomass $B_{t+1}$ of a stock in the year t+1 can be expressed as the biomass in year *t* ($B_{t}$) plus the surplus production in year *t* ($Y_{t}$), minus the harvested amount in year *t* ($C_{t}$). This can be formulated as follows [16]:

(9) $$B_{t+1}=B_{t}+Y_{t}-C_{t}=B_{t}+rB_{t}\left(1-\frac{B_{t}}{K}\right)-C_{t}$$

where *r* represents the intrinsic rate of increase and *K* denotes the carrying capacity.

In fisheries stock assessment, CPUE (*A*) is typically employed as indices of resource abundance and is directly associated with biomass through the catchability coefficient (*q*):

(10) $$A_{t}=B_{t}q$$

By integrating Formula (9) and Formula (10), the following expression can be derived:

(11) $$A_{t+1}=A_{t}+A_{t}r\left(1-\frac{A_{t}}{Kq}\right)-C_{t}q$$

In the AMSY, assessing the relative exploitation status of a fish population may be achieved without obtaining the absolute values of $C_{t}$, $B_{t}$, *K*, and *q*. Instead, the relative yield $C_{qt}$ can be substituted for $C_{t}q$, and the relative carrying capacity $K_{q}$ can replace Kq; then, Equation (11) can be transformed as follows (Froese et al., 2019 [16]):

(12) $$C_{qt}=A_{t}+A_{t}r\left(1-\frac{A_{t}}{K_{q}}\right)-A_{t+1}$$

In the Schaefer surplus production model [47], where $F_{MSY}=r/2$ and $B_{MSY}=K/2$, MSY can be obtained as rK/4. Consequently, the transformed ${MSY}_{q}$ according to Equation (12) can be expressed as $rK_{q}/4$ [48]. ${MSY}_{q}$ represents the MSY corresponding to the relative catch $C_{q}$; therefore, the value of $C_{q}{MSY}_{q}$ is equivalent to the value of the relative yield C/MSY. Similarly, the value of $A_{t}/K_{q}$ is equivalent to the value of $B_{t}/K$, and the calculation formula for the fishing mortality rate (*F*) as $C_{t}/B_{t}$ can be expressed using the value of $C_{qt}/A_{t}$. The biological reference point $F/F_{MSY}$ can be computed using the following formula [16]:

(13) $$\frac{F}{F_{MSY}}=\frac{\frac{C_{qt}}{A_{t}}}{r/2}=\frac{2C_{qt}}{rA_{t}}\;$$

The prior for parameter $K_{q}$ in the model can be obtained through the expression $A_{t}/\left(B_{t}/K\right)$, while the value of parameter $B_{MSY\_q}$ is derived from $K_{q}/2$. Generating multivariate lognormal random samples for the parameter combination $r-K_{q}$ through the utilization of the variance–covariance matrix (VCM):

(14) $${VCM}_{log\left(r\right),log\left(K_{q}\right)}=\left\{\left.\begin{array}{cc} \sigma_{log\left(r\right)}^{2} & {cov}_{log\left(r\right),log\left(K_{q}\right)}\\ {cov}_{log\left(r\right),log\left(K_{q}\right)} & \sigma_{log\left(K_{q}\right)}^{2} \end{array}\right\}\right.$$

where cov represents the covariance between two parameter distributions, and $\sigma^{2}$ denotes the variance.

Based on Markov Chain Monte Carlo (MCMC), a filtering method is employed to aid in the identification of $r-K_{q}$ parameter combinations that align with CPUE data and prior information. Multiple tests are conducted for each $r-K_{q}$ combination, varying the random error settings for surplus production and CPUE. Combinations that fail to pass the MCMC filter in multiple tests are subsequently excluded from further consideration. Specifically, during the model run, the AMSY model randomly selects combinations of *r* and *K*_q_ of the prior distribution to estimate the catch that can match the resource abundance along with the two prior information inputs. It then applies a series of filters to eliminate any *r*-*K*_q_ combinations that produce negative catches or do not meet the actual exploitation value. Finally, the ‘viable’ *r*-*K*_q_ pairs are used to calculate ${MSY}_{q}$. The AMSY model requires time-series CPUE data, prior distribution range for *r*, and prior information on the relative population abundance level $\left(B_{t}/K\right)$. The AMSY model recommends, in the absence of specific studies, setting the prior distribution range for the population parameter *r* based on the resilience grading table from Fishbase (www.fishbase.org, accessed on 12 October 2023). The Japanese sardine is a small pelagic fish and has a relatively high resilience for population recovery. In our study, according to the assessment results of Yang et al. [28], we set the prior information for *r* as the uniform distribution U (0.6, 1.5). For $\left(B_{t}/K\right)$, the prior setting for this parameter is determined based on the table provided in the AMSY user guide [16]. In 2014, the resource level of Japanese sardines in the NPO was approximately at a moderate level compared to the initial resource amount; hence, we set the $\left(B_{t}/K\right)$ for 2014 at about half [16]. In addition to the abundance index of three seasons, we also calculated the average of the three indices to obtain the fourth abundance index series and facilitated stock assessment for comparative analysis.

### 2.5. Stock Assessment Performance When Fitting the Spatio-Temporal Index and Conventional Model-Based Index

We also investigated the predictive performance of the AMSY method using the spatio-temporal index and the conventional model-based index. A cross-validation test was facilitated to address the comparison. The difference between the model-fitted index and the observed index for a given year was quantified using relative error (*RE*). The formula for *RE* was as follows:

(15) $${RE}_{y}=\frac{{CPUE}_{y}^{pred}-{CPUE}_{y}^{obs}}{{CPUE}_{y}^{obs}}$$

where ${CPUE}_{y}^{pred}$ represents the fitted value of abundance for Japanese sardines in the *y*th year and ${CPUE}_{y}^{obs}$ is the spatio-temporal index/conventional model-based index for the *y*th year. We also calculated the average absolute relative error (*ARE*) to compare model predictive performance based on spatio-temporal and conventional model-based indices using the following equation:

(16) $$ARE=\frac{\sum_{y=1}^{n_{y}}\left|{RE}_{y}\right|}{n_{y}}$$

$n_{y}$ represents the number of years of study data. The data used in this paper spans from 2014 to 2022, so $n_{y}$ is 9.

## 3. Results

### 3.1. Model Fitting and Diagnostics

The final gradient of all fixed effects in the seasonal spatio-temporal model of Japanese sardines in the NPO was less than 10^−4^. Additionally, the Hessian matrix for the second derivatives of the negative log-likelihood was positive definite, which proved that the model is convergent and also showed that the fitting performance of our seasonal spatio-temporal model was good and could accurately estimate the standardized abundance index for Japanese sardines. Importantly, the results of autocorrelation for estimating the spatio-temporal fluctuation arising across seasons and years were $\rho_{n\varepsilon }$ = −0.172 and $\rho_{w\varepsilon }$ = −0.449. Specifically, the coefficient $\rho_{n\varepsilon }$ = −0.172 indicates weak negative autocorrelation of spatial variation between seasons. On the other hand, $\rho_{w\varepsilon }$ = −0.449 reflects moderate negative autocorrelation of temporal variation across years, implying that high abundance years are often followed by low abundance years, and vice versa. The presence of these coefficients allows the model to make effective predictions in areas lacking direct sampling data. By leveraging data from other seasons and years, the model can generate reasonable abundance estimates for regions without data.

### 3.2. Standardized Seasonal Relative CPUE

**Figure 2:**
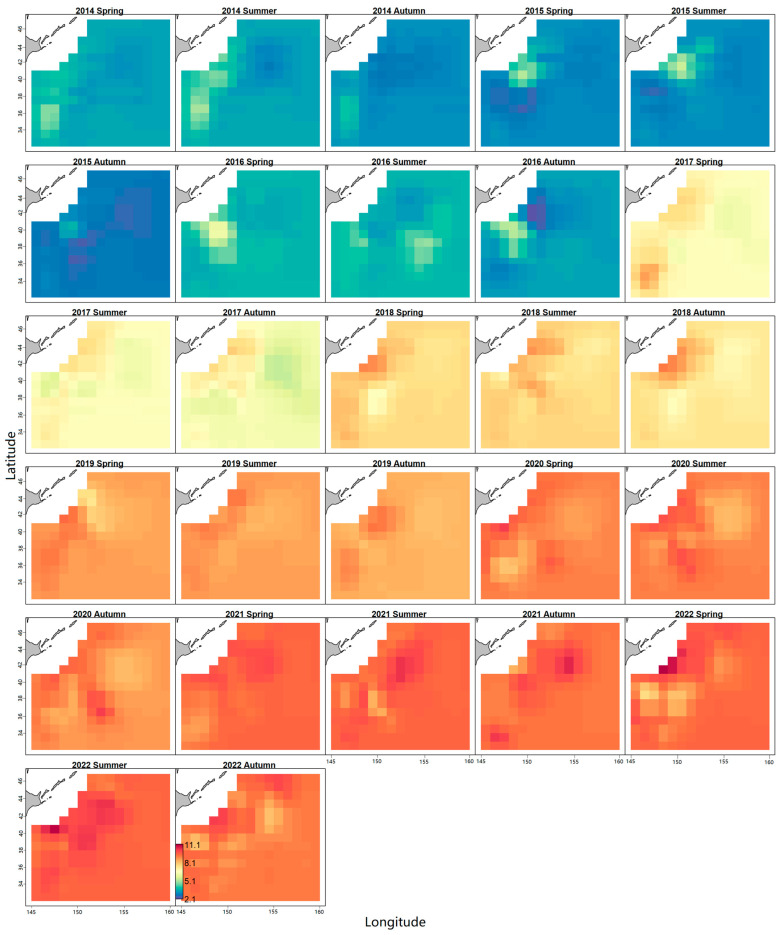
The predicted seasonal log densities of Japanese sardines from 2014 to 2022 in the NPO. The units is kg/km^2^. A clear color legend is located in the bottom left corner of the final image, with density values increasing progressively from green to yellow and then to red.

**Figure 3:**
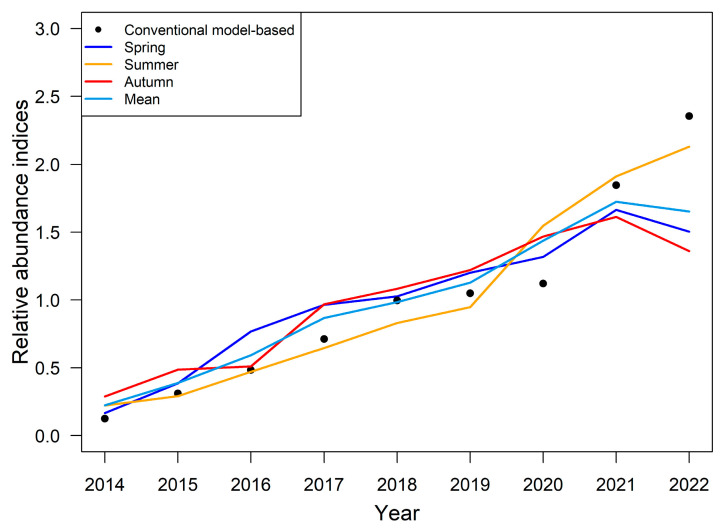
The relative abundance of conventional model-based indices for three seasons and the mean abundance indices for three seasons.

We demonstrated the seasonal changes in the log density of Japanese sardines from 2014 to 2022. Figure 2 shows that over time, the apparent color changes from blue to yellow and finally to red, indicating an increase in the abundance of Japanese sardine stock. Note that compared to 2021, the panel for 2022 showed more yellow areas and a decrease in resource abundance (Figure 2). We then obtained annual abundance indices for Japanese sardines in the NPO for three seasons (Figure 3), evaluated by the seasonal spatio-temporal model. Meanwhile, the mean yearly abundance indices of the three seasons were calculated for further stock assessment research. Figure 3 reveals that from 2014 to 2021, the four resource abundance data showed similar trends, and all of them gradually increased. However, the spatio-temporal index showed lower temporal variation than the conventional model-based index. In 2022, except for the summer resource abundance, which continued to increase, the other three resource abundance indices all decreased. In 2022, the conventional model-based indices increased significantly and reached the maximum value, while the resource abundance index estimated based on the seasonal spatio-temporal model we constructed tended to be stable in that year (Figure 3). The spatio-temporal index judges spike observations as “outliers” when compared to the simpler average used by the conventional model-based indices. Consequently, these samples hold less statistical influence in the spatio-temporal index than the conventional model-based index. This observation supports the theory proposed by Thorson et al. [39].

**Figure 4:**
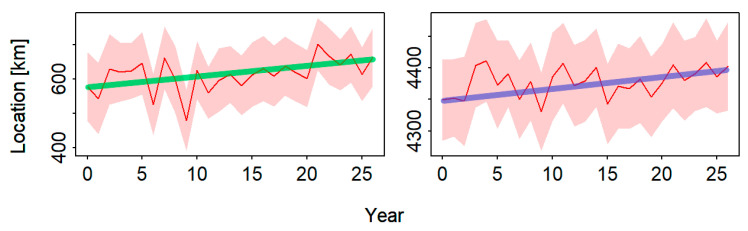
The COG variation of Japanese sardine distribution patterns in eastward (**right**) and northward (**left**) directions from Spring 2014 to Autumn 2022. The numbers in the horizontal ordinate represent Spring 2014, Summer 2014, Autumn 2014, Spring 2015, Summer 2015, …, Spring 2022, Summer 2022, and Autumn 2022, respectively. The colored diagonal lines in each figure represent the trend lines of COG changes.

Regarding the COGs of Japanese sardine abundance, an eastward and northward trend between 2014 and 2022 was observed (Figure 4). Specifically, the COGs of Japanese sardine stock had a significant variation in the eastward direction (Kendall’s tau = 0.351, *p* = 0.011), while the change in COGs for Japanese sardine stock in the northward direction was non-significance (Kendall’s tau = 0.266, *p* = 0.055). The variability ranges of COGs in the eastward and northward directions in the period 2014–2022 were 97 km and 60 km, respectively.

### 3.3. The Results of Stock Status for Japanese Sardines Based on AMSY

**Figure 5:**
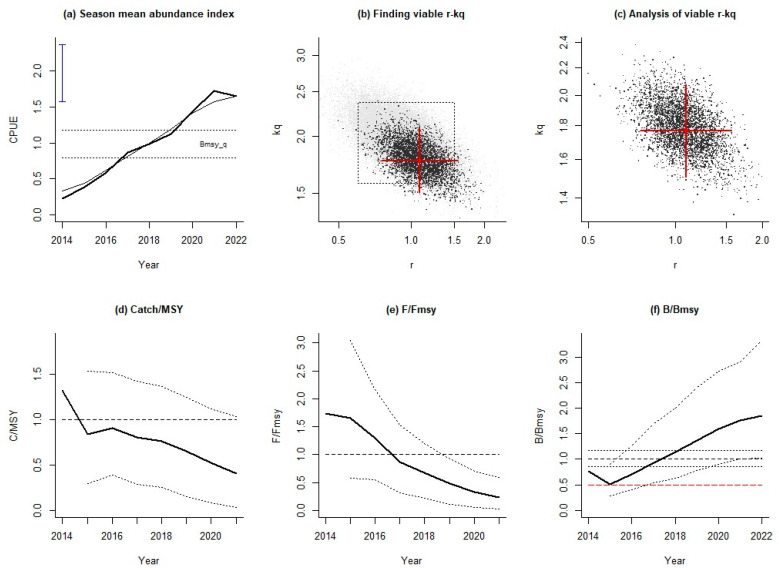
Assessment results of AMSY for Japanese sardines in the NPO using the mean abundance indices of three seasons. (**a**) The trend of yearly standardized CPUE based on the VAST method. Two dotted horizontal lines represent the prior bounds for the CPUE level that correspond to *B*_MSY_, and the blue vertical line with bars at the end shows the prior stock size *B*/*B*_0_. (**b**) The tested *r*-*k*_q_ pairs (grey dots) and ‘viable’ *r*-*k*_q_ pairs (black dots). (**c**) The magnified area occupied by the ‘viable’ *r*-*k*_q_ pairs and the most likely *r*-*k*_q_ pair at its center. (**d**) The trend of the median relative catch (bold curve). The two dotted curves represent the approximate 95% confidence limits. (**e**) The *F*/*F*_MSY_ trend (bold curve). The dotted curves represent the approximate 95% confidence limits. (**f**) The trend of CPUE expressed as *B*/*B*_MSY_ (bold curve). The two dotted curves are the corresponding approximate 95% confidence limits. The dashed red line represents the stock size below which recruitment may be impaired.

According to the results of AMSY obtained using the average abundance index of three seasons, the relative catch of Japanese sardines showed a trend of first decreasing, then increasing, and then decreasing from 2014 to 2022, and the catch was less than MSY after 2015, i.e., *C*/MSY was less than 1 (Figure 5d). The grey dots in Figure 5b indicate the *r*-*k*_q_ pairs that were tested by AMSY. The black dots represent ‘viable’ *r*-*k*_q_ pairs that passed the AMSY filters. Figure 5c shows the magnified area occupied by the viable *r*-*k*_q_ pairs, shown again in black. The fishing pressure showed a gradually decreasing trend, and 2017 was the cut-off point of fishing intensity (Figure 5e). Before 2017, the fishing intensity was too large, *F*/*F*_MSY_ was greater than 1, and the resource was in an overfishing state; after 2017, it was less than 1 and the resource was healthy. The relative population biomass *B*/*B*_MSY_ of Japanese sardines decreased first and then increased, and from 2014 to 2017, the relative resource was less than 1, indicating a poor stock status (Figure 5f). After 2018, Japanese sardine stocks returned to a healthy status (*B*/*B*_MSY_ > 1). It is noted that in 2015, the *B*/*B*_MSY_ value was less than 0.5, indicating that the recruitment of Japanese sardine resources had been hindered.

**Figure 6:**
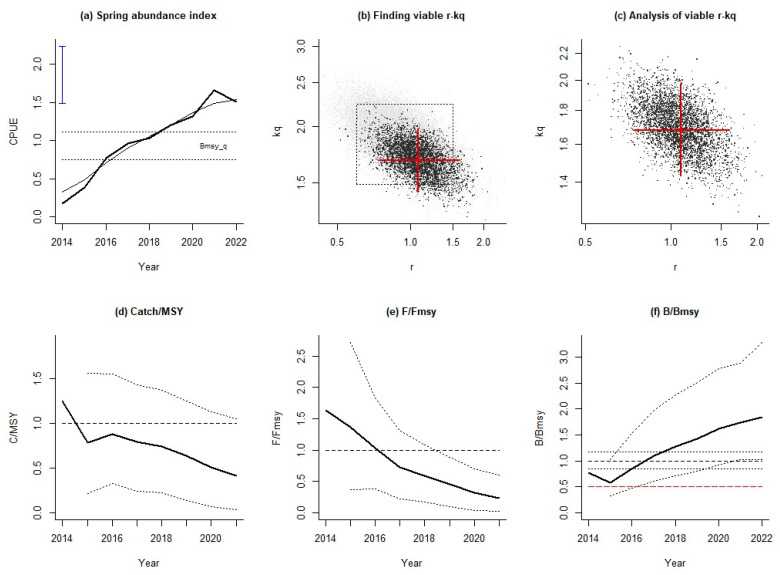
Assessment results of AMSY for Japanese sardines using the spring abundance index.

According to the stock assessment results using the spring resource abundance index, the trend of relative catch was consistent with the results based on the mean abundance indices of three seasons (Figure 6d). Fishing pressure in 2014–2018 was lower than results based on average abundance indices of three seasons, while the opposite was true in recent years. After 2017, the fishing intensity was also lower than the overfishing value (Figure 6e). The relative biomasses of Japanese sardines in the spring of 2014–2022 were generally higher than those using the average resource abundance index, and the resources were in good health after 2018, and there were no years with relative biomass less than 0.5 (Figure 6f).

**Figure 7:**
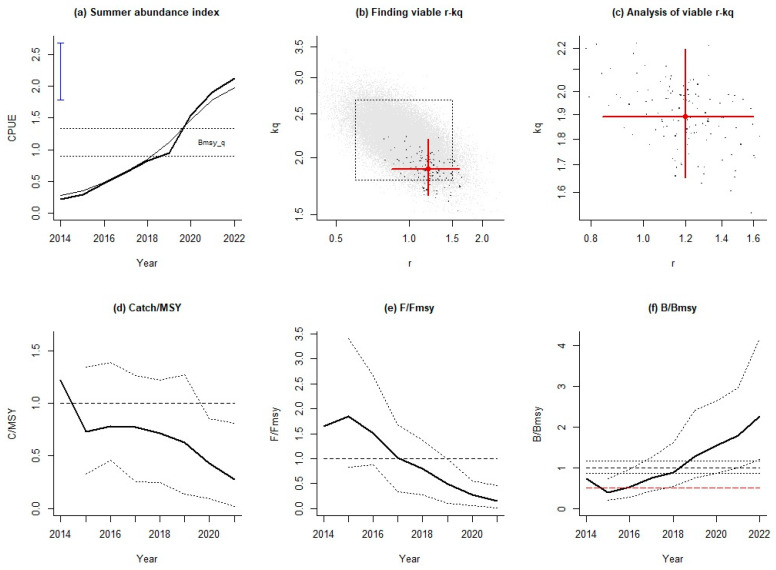
Assessment results of AMSY for Japanese sardines using the summer abundance index.

The results of AMSY using the summer resource abundance index indicated that the relative catch showed a fluctuating downward trend, similar to other periods (Figure 7d). For fishing pressure, the results based on summer abundance were higher than those for spring and average abundance in the early part of the study period, but in the last three years, the trend reversed (Figure 7e). Correspondingly, summer relative biomasses were lower than those in other periods in the early part of the study period, but in the recent period 2020–2022, the relative biomass was larger than those in other periods (Figure 7f).

**Figure 8:**
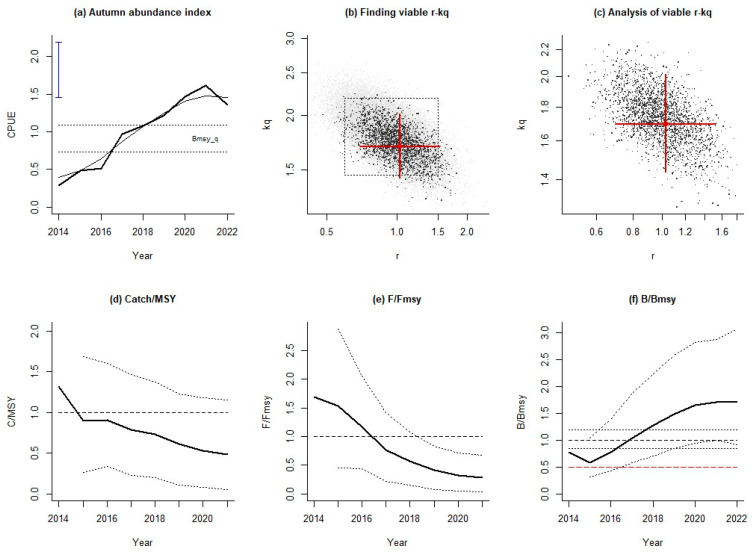
Assessment results of AMSY for Japanese sardines using the autumn abundance index.

**Figure 9:**
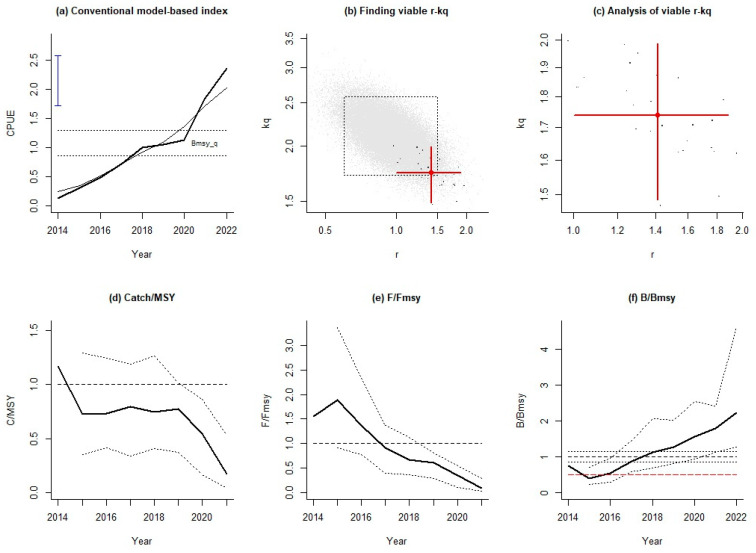
Assessment results of AMSY for Japanese sardine using the conventional model-based abundance index.

From the stock assessment results based on the autumn abundance, the resource was overfished in 2014–2016, as both the fishing pressure and the relative stock biomass were out of line with the accepted levels (Figure 8e,f). Japanese sardine stocks recovered to a healthy state after 2017. Notably, the autumn resource abundance index showed a significant decline in 2022 (Figure 8a), and compared with the growth trend of relative biomass in other periods, the relative biomass in autumn tended to be flat, and those in 2021 and 2022 were also smaller compared to other periods. In general, during the study period, the fishing pressure in autumn was generally higher than in spring and summer, and the relative biomass values in the past two years were smaller than those in other periods. Stock assessment based on the conventional model-based abundance index was also conducted to compare fitting results based on the spatio-temporal index (Figure 9).

**Figure 10:**
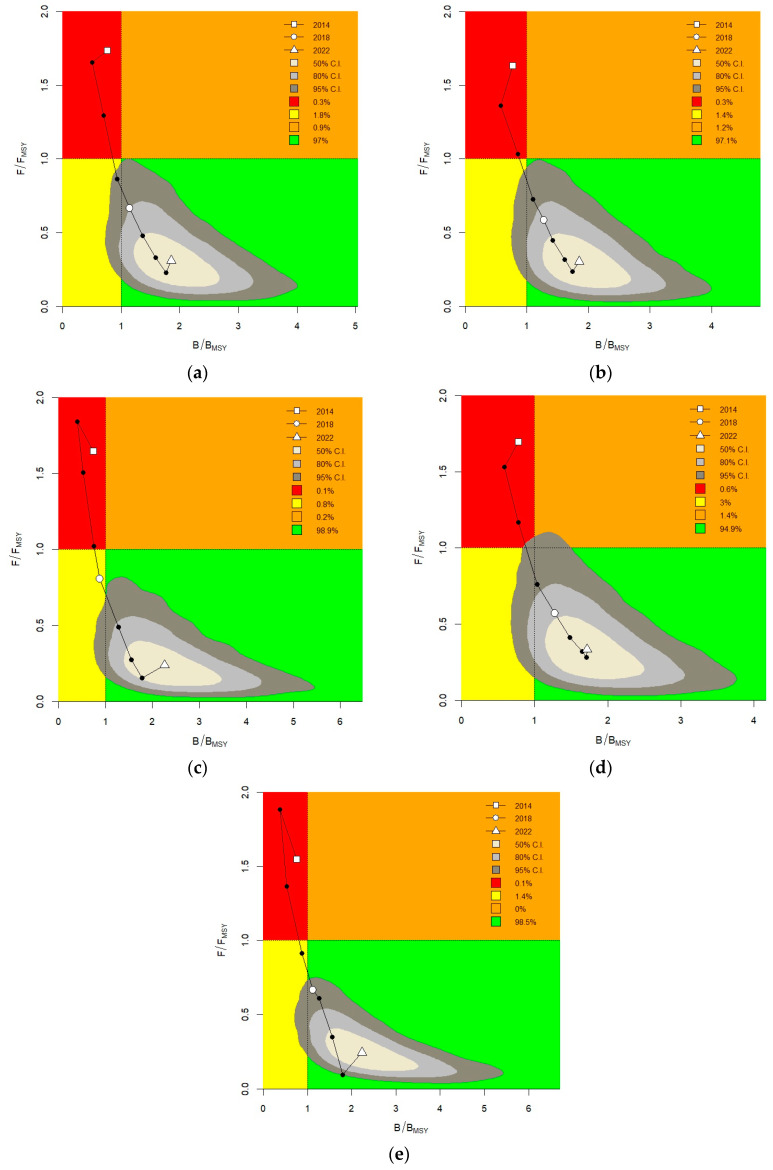
Kobe plot of AMSY for Japanese sardines based on abundance indices of five time series. The green area represents a sustainable fishery status, the orange area indicates that the resource is overfished but not currently experiencing overfishing, the red area signifies that the resource has been overfished, and the yellow area denotes that the resource is overfished but not currently undergoing overfishing. (**a**) Mean abundance index. (**b**) Spring abundance index. (**c**) Summer abundance index. (**d**) Autumn abundance index. (**e**) Conventional model-based abundance index.

Figure 10 presents the trajectories of fishing pressure (*F*/*F*_MSY_) and relative biomass (*B*/*B*_MSY_) of AMSY results using spring, summer, autumn, average abundance index, and conventional model-based index. The overall trend of the five Kobe plots is consistent, indicating that Japanese sardine stock in the NPO is recovering from overfishing (mainly in the red area in the early period) to healthy (in the green area in the later period). The current stock status (2022) is in the green area, and the resources are in a healthy state. However, the *B*/*B*_MSY_ values are quite different, and the values in autumn are clearly lower than those in other periods. Results based on the spatio-temporal index (Figure 10a) were more conservative (*B*/*B*_MSY_ values are smaller) than results based on the conventional model-based index (Figure 10e).

### 3.4. Comparison of Model-Fitted Performance Under Conventional Model-Based Index and Spatio-Temporal Index

**Figure 11:**
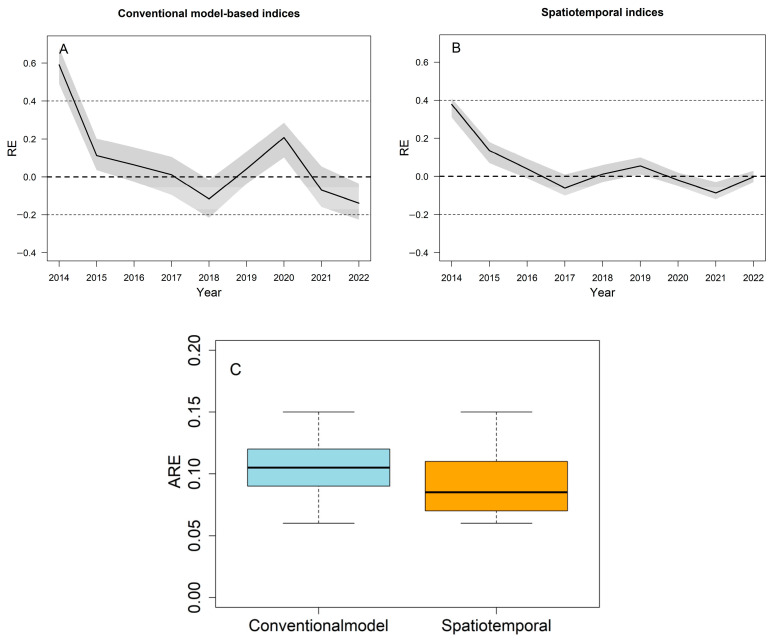
The RE and ARE of predicted indices based on the AMSY model using spatio-temporal and conventional model-based indices. The grey shadow part in (**A**,**B**) represents the 95% confidence interval, while the different colors in (**C**) represent the use of different standardized models.

The AMSY model was fitted using the conventional model-based index and the estimated spatio-temporal index. The estimated relative abundance indices from the AMSY method were very close to the spatio-temporal index from 2014 to 2022, while the predicted results when using the conventional model-based index had some exceptions in some periods, e.g., 2020 and 2022 (Figure 9a). Correspondingly, the spatio-temporal index had a smaller RE and a narrower 95% interval compared to the conventional model-based index (Figure 11A,B). Regarding the ARE, the spatio-temporal index also exhibited smaller ARE values that were closer to zero (Figure 11C). This indicates that the AMSY model using the spatio-temporal index outperformed the model fitted with the conventional model-based index in predicting stock abundance indices.

## 4. Discussion

Seasonal migration of fish stock is a significant source of uncertainty in fishery stock assessment [20,32]. Previous studies rarely integrated precise migration information into stock assessment models due to model complexity, viewing the research area as a whole, which negatively impacted fisheries management [49,50]. Japanese sardine, a key species in the NPO with substantial economic and ecological value [51,52], exhibits distinct seasonal migration influenced by physiological needs, genetics, and environmental factors [30,53]. Therefore, to promote the sustainable development of Japanese sardine fishing and conserve marine biodiversity, this migration should be incorporated into management strategies. We constructed a seasonal spatio-temporal model for Japanese sardines in the NPO in this study to standardize the abundance indices of three seasons (spring, summer, and autumn) and calculate their average. Then, it was used as model input in the data-limited method to obtain the refined assessment results and provide a scientific basis for the effective management of this stock. In addition, the NPFC began to work on stock assessment of Japanese sardine fishery and set up relevant working groups to carry out CPUE standardization [31]. Therefore, this study reflects our commitment to international fishery compliance and lays the foundation for effective management recommendations.

Spatio-temporal models are increasingly used to estimate fishery resource density, demonstrating greater statistical efficiency than spatial hierarchical models in some cases [54,55]. These models evaluate autocorrelation among nearby locations and timeframes, assuming that densities across diverse locations have unique predicted values based on habitat covariates and spatial components, with closer sites exhibiting greater similarity [56]. However, conventional model-based estimators assume a constant mean for a specific stratum, leading to greater sensitivity to outliers [20]. Given the spatio-temporal heterogeneity in Japanese sardine fishery data in the NPO, our study demonstrates how to use a seasonal spatio-temporal model to address data imbalances and derive an accurate resource abundance index. This study is the first to apply such a model to China’s distant-water fishery. Previous models typically addressed only one type of interannual or seasonal variation [35,57]. In contrast, our model incorporates year and season effects along with autocorrelated season–year effects for intercept and spatial change, accounting for the full correlations between year and season [58]. Therefore, it effectively addresses the imbalance in fishery-dependent data for Japanese sardines.

Accurate estimation of the resource abundance index is crucial for the sustainable utilization and scientific management of fishery resources [59]. Our results indicate that the standardized abundance index of Japanese sardines in the NPO gradually increased from 2014 to 2021, suggesting resource recovery. In 2022, there was a slight decrease in spatio-temporal abundance indices, except in the summer (Figure 3). However, the conventional model-based index showed a significant increase in 2022, reaffirming Thorson et al.’s [39] assertion that spatio-temporal indices exhibit less temporal variation and are less affected by outliers. Shi et al. [60] applied the General Linear Model (GLM), the Generalized Linear Mixed Model (GLMM), and the spatio-temporal GLMM (VAST) to standardize the abundance indices of Japanese sardines, yielding trends consistent with our findings. In terms of spatial variations, Japanese sardines exhibit significant seasonal variations, with high-density areas moving eastward from spring to autumn (Figure 2). The COGs of Japanese sardine fishery generally moved in the northeast direction, with significant movement eastward (*p* < 0.05) (Figure 4). Therefore, using the seasonal spatio-temporal model to predict and estimate the spatio-temporal distribution pattern has important guiding significance for fishery production, refined fishery management, and the protection of the NPO ecosystem. This allows fishermen to identify optimal fishing grounds and enables managers to focus on low-density areas. In addition, although previous studies on Japanese sardine population dynamics often focused on the 1990s or early 20th century [61,62], our study is based on the latest Chinese lighting purse seine fishery data, making our management recommendations more time-sensitive.

Beyond the applications highlighted in this study, the seasonal spatio-temporal model is effective in scientific resource surveys with reduced stations or incomplete strata due to severe weather, ship failures, or funding constraints. It can estimate resource abundance in adjacent seasons or unsurveyed regions based on existing survey data [39]. With China’s increasing emphasis on the protection of marine fishery resources, the State Council issued the “Opinions on Promoting the Sustainable and Healthy Development of Marine Fisheries” (Guofa (2013) No. 11), emphasizing the importance of fishery resources surveys and requiring a comprehensive survey of fishery resources to be conducted every five years, with perennial monitoring and assessment, focusing on the investigation of important fishery resources such as endangered species, aquatic germplasm, and important fishery waters such as spawning grounds for economic organisms, river estuaries and the South China Sea [63]. The seasonal spatio-temporal model can enhance these investigations by standardizing the resource abundance index of target species. It can also infer interannual variations in species phenology when data are imbalanced [39]. This study applied the model to the Japanese sardine, a small pelagic fish in the NPO that exhibits resource distribution and seasonal migration that is highly sensitive to marine environmental changes [52,62,64]. Therefore, how to integrate marine environmental factors into the seasonal spatio-temporal model is the key direction of our future research. Additionally, it should be noted that despite these advantages, predictions of abundance using a spatio-temporal model may be skewed if (1) extensive sections of the population domain are devoid of fishery data, thus going unnoticed, and (2) the patterns in the unseen areas systematically diverge from the ones observed [65].

The data-limited AMSY method was applied to assess Japanese sardine stocks in the NPO, demonstrating effectiveness in managing data-limited fisheries by estimating productivity and relative population size [16]. AMSY integrates resource abundance indices, prior information on growth rates (r), and relative resource sizes to estimate biological reference points such as *F*_MSY_, *F*/*F*_MSY_, and *B*/*B*_MSY_. The prior distribution for *r* is typically based on biological parameters or empirical formulas [11]. Froese et al. [16] recommended using the length-based model (LBB) to evaluate the parameter *B*_t_/*K* based on body length frequency data. Based on the surplus production model, the AMSY model randomly selects the combination of *r* and *K*_q_ to estimate the catch that can match the resource abundance and the two prior information and uses a series of filters to eliminate the *r*-*K*_q_ combination that produces negative catch or does not meet the actual exploitation value, so as to improve the accuracy and credibility of stock status estimation [16]. Our assessment utilized a conventional model-based index and four indices from the seasonal spatio-temporal model, revealing that Japanese sardine resources have been gradually recovering from 2014 to 2022 (Figure 2 and Figure 3). In 2022, there was a 94.8–99% probability of sustainable exploitation, a 0.7–3.1% chance of recovery from depletion, and a 0.1–0.6% risk of being overfished (Figure 10). Yang et al. [28] evaluated the stock status of Japanese sardines based on Catch-MSY and LBB models and found that the current Japanese sardine resources were in a healthy state, which was consistent with the results of this study. Wang et al. [66] applied an extension of the Catch-MSY model to facilitate stock assessment of Japanese sardines during 1980–2017 and found that the stock status of Japanese sardines had collapsed, which was different from our results, probably because the Catch-MSY model was sensitive to catch data, and the study period was during a low stage of Japanese sardine catches. Our model comparison showed that the spatial-temporal index had lower relative errors (REs) and absolute relative errors (AREs), along with higher predictive accuracies (Figure 11). The Kobe plot shows that the stock assessment results of the spatio-temporal index are more conservative than that of the conventional model-based index, which is consistent with the results of Falson et al. [67]. Abundance index bias and the prior information of relative stock size are important factors affecting the accurate estimation of AMSY. Abundance index bias is caused by (1) the continuous increase in fishery efficiency (e.g., improvement in fishing experience and fishing techniques) to capture specific species and (2) the “hyperstability” and “hyperdepletion” of commercial fishing resources abundance. The former will lead to an overestimation of resource biomass and an underestimation of fishing pressure, while the latter will lead to excessive pessimism about the information on stock status [16]. To solve the problem above, we believe that the external influence factors can be removed through abundance index standardization so as to reduce the bias and improve the accuracy of the model results. Resource abundance index standardization has been widely used in fishery resources research [68,69,70]. For the determination of prior information of relative population size, it can be solved using a length-based model, which has been confirmed in studies by Zhang [63] and Froese et al. [16]. Zhang [63] pointed out that the evaluated results of parameters such as MSY_q_ and *F*_MSY_ are sensitive to different *r* prior distributions, while the *B*/*B*_MSY_ and *F*/*F*_MSY_ are sensitive to the lower limit setting of *B*_t_/*K* prior distribution ranges. In addition, the simulation test of the AMSY model shows that the relative fishing pressure *F*/*F*_MSY_ estimated by the model has a large uncertainty and therefore needs to be used with caution, while the estimation of the relative biomass *B*/*B*_MSY_ has good performance, which can be used for management recommendations in data-limited fisheries [16]. However, under data-limited conditions, the fishery stock assessment methods are a tactic of last resort. The uncertainty caused by missing information for the validation of the AMSY model is primarily reflected in several aspects. Firstly, missing data may lead to inaccurate estimates of key parameters, thereby affecting the biological reliability of the model. Secondly, the lack of information weakens the model’s ability to predict future catch and population sizes. Additionally, the absence of complete data makes it difficult to validate the consistency of model outputs with actual observed data. Therefore, in the actual fishery stock assessment and management, on the one hand, it is necessary to collect as much data as possible and to meet different assessment methods. On the other hand, it is necessary to set the distribution range of key parameters with reference to reliable evaluation results.

Our results also found that, in terms of assessment results for three seasons, the resource abundance index in summer continued to increase (Figure 3), leading to the most optimistic stock status in summer compared with spring and autumn (Figure 10). Autumn has a more volatile resource abundance index and the most pessimistic estimates of stock status, which provides important information for managers to refine their fisheries resource management. Autumn is the season when Japanese sardines migrate from feeding grounds to spawning grounds. Therefore, in order to ensure the successful recruitment of Japanese sardines, autumn fishing intensity needs to be managed, such as by reducing autumn quotas or vessel numbers, which plays an important role in the protection of Japanese sardine stocks and the ecosystem of the NPO.

Japanese sardine resources in the NPO have experienced many drastic fluctuations since 1950, with a low level of average catch of around 150 thousand tons in 1950–1970, a rapid increase in catches in 1970–1990, and a peak of about 5.5 million tons in 1988, followed by a sharp decline in sardine catches, reaching less than 100 thousand tons in 2000. The catch from 2000 to 2022 gradually increased, and in 2020, the production exceeded 1 million tons. Over the past 70 years, Japanese sardine stocks have fluctuated considerably [62]. The increase in catches in the 1970s was attributed to intensive fishing by several countries [51]. Watanabe et al. [71] pointed out that the decline in Japanese sardine resources in the 1990s was caused by the low survival rate of the recruitment population, and this recruitment failure may be due to the sudden increase in SST caused by climate change [72]. Therefore, the variation in Japanese sardines is a cumulative influence of many factors, including fishing intensity, climate change, and resource replenishment. It is more optimistic that Japanese sardine stock in the NPO is currently in the recovery stage and has not been overfished. The recovery of the Japanese sardine population in the NPO can be attributed to several pivotal factors. Firstly, the establishment of the NPFC in 2015 marked a significant advancement in the scientific management and assessment of fisheries resources. This initiative concentrated on the primary fishing method for Japanese sardines, specifically lighting purse seine fisheries, which effectively mitigated fishing pressure in the region. Secondly, alterations in climatic and oceanographic conditions, including variables such as sea surface temperature (SST), sea surface height (SSH), and chlorophyll-a concentration (Chla), have fostered a more conducive environment for the Japanese sardine population. Lastly, Japanese sardines are categorized as small pelagic fish characterized by rapid growth rates and notable resilience to population declines. These inherent biological traits, combined with reduced fishing pressure, facilitate a more expedient recovery of the Japanese sardine population. The recovery of this resource is important for the conservation of marine ecosystems in the NPO, and we all know that Japanese sardines play a role in the food web system. Japanese sardines mainly feed on phytoplankton, copepods, and diatoms and are preyed on by large fish such as tuna [73], so the recovery of Japanese sardine resources not only ensures that the marine food web is more robust but may also play a role in protecting other economic species.

Based on this study, we propose the following management recommendations: (1) For fishermen, Japanese sardine is prioritized by the NPFC, and quota-based fishing is becoming essential. Efficient fishing timing is crucial to minimize economic and ecological costs. Given the higher abundance of Japanese sardines in the summer and lower abundance in the autumn, fishermen should reduce vessel numbers in autumn and increase them in spring and summer. This approach enhances profitability, conserves fuel, and reduces carbon emissions, benefiting the NPO’s ecological environment. (2) For fisheries managers, our findings can guide the allocation of fishing efforts throughout different seasons. They should increase efforts during high-density seasons and decrease them in less favorable periods, supporting the recovery and sustainable use of resources. Additionally, managers should enhance ecosystem-based fisheries management, integrating seasonal variations into decision-making and encouraging member countries to share data for better monitoring and assessment of Japanese sardine resources. This international cooperation can address resource management complexities and promote consistent global standards. (3) For researchers, many fisheries lack comprehensive data for stock assessment, similar to the Japanese sardine situation. Our study offers a new perspective for managing species with limited data. Seasonal spatio-temporal models can estimate resource abundance over consecutive years, even if scientific surveys are interrupted. Although this study indicates a healthy status for Japanese sardines, caution is advised to prevent repeating past resource declines.

## 5. Conclusions

The purpose of this paper was to construct a framework for refined fishery stock assessment based on a seasonal spatio-temporal model and data-limited method, taking Japanese sardines in the NPO as an example. Firstly, the resource abundance index of Japanese sardines in three seasons was standardized based on the seasonal spatio-temporal model, which can fully consider the imbalance in fishery data so as to obtain an accurate and ecologically significant abundance index. Then, the resource abundance index was used as the AMSY model input to carry out the seasonal stock assessment, understand the status of Japanese sardine resources in each season, and put forward targeted management suggestions. The framework of this study can not only provide technical support for the refined management of distant-water fishery resources but also provide new ideas for the investigation and research of offshore fisheries. Summarizing the results of our study, the abundance index of Japanese sardine resources showed an upward trend during the study period, the population COGs generally moved in the northeast direction, and the upward movement in the east was significant (*p* < 0.05). The performance of resource evaluation fitting using a spatio-temporal index is better than that using the conventional model-based index. At present, Japanese sardine stock is in a healthy status and is gradually recovering. Nonetheless, given the worrying fluctuations in Japanese sardine stock in the NPO over the past 70 years, we advocate caution in the exploitation of Japanese sardine stock.

## Data Availability

Data will be made available on request.

## References

[1] Cadrin S.X., Dickey-Collas M. (2015). Stock assessment methods for sustainable fisheries. ICES J. Mar. Sci..

[2] Suncls A., Cai I. United Nations Convention on the Law of the Sea; Proceedings of the United Nations 1982. https://www.un.org/depts/los/convention_agreements/texts/unclos/closindx.htm.

[3] Brooks E.N., Powers J.E., Cortés E. (2010). Analytical reference points for age-structured models: Application to data-poor fisheries. ICES J. Mar. Sci..

[4] Ricard D., Minto C., Jensen O.P., Baum J.K. (2012). Examining the knowledge base and status of commercially exploited marine species with the RAM legacy stock assessment database. Fish Fish..

[5] Neubauer P., Thorson J.T., Melnychuk M.C., Methot R., Blackhart K. (2018). Drivers and rates of stock assessments in the United States. PLoS ONE.

[6] Newman D., Berkson J., Suatoni L. (2015). Current methods for setting catch limits for data-limited fish stocks in the United States. Fish. Res..

[7] Pons M., Cope J.M., Kell L.T. (2020). Comparing performance of catch-based and length-based stock assessment methods in data-limited fisheries. Can. J. Fish. Aquat. Sci..

[8] Shi Y.C., Fan W., Zhang H., Zhou W.F., Tang F.H., Wu Z.L., Cheng T.F., Zhao G.Q., Zhang X.M. (2021). Review on stock assessment methods applicable to data-limited fisheries. J. Fish. Sci. China.

[9] MacCall A.D. (2009). Depletion-corrected average catch: A simple formula for estimating sustainable yields in data-poor situations. ICES J. Mar. Sci..

[10] Martell S., Froese R. (2013). A simple method for estimating MSY from catch and resilience. Fish Fish..

[11] Zhang K., Zhang J., Xu Y.W. (2018). Application of a catch-based method for stock assessment of three important fisheries in the East China Sea. Acta Oceanol. Sin..

[12] Arnold L.M., Heppell S.S. (2015). Testing the robustness of data-poor assessment methods to uncertainty in catch and biology: A retrospective approach. ICES J. Mar. Sci..

[13] Rudd M.B., Thorson J.T. (2018). Accounting for variable recruitment and fishing mortality in length-based stock assessments for data-limited fisheries. Can. J. Fish. Aquat. Sci..

[14] Baibbat S.A., Pons M., Chattou E.M.A. (2019). A length based assessment for Atlantic Bonito (*Sarda sarda*) exploited in the Moroccan Atlantic Coast. ICCAT.

[15] Liang C., Xian W., Liu S., Pauly D. (2020). Assessments of 14 Exploited Fish and Invertebrate Stocks in Chinese Waters Using the LBB Method. Front. Mar. Sci..

[16] Froese R., Winker H., Coro G., Demirel N., Tsikliras A.C., Dimarchopoulou D., Scarcella G., Palomares M.L.D., Dureuil M., Pauly D. (2019). Estimating stock status from relative abundance and resilience. ICES J. Mar. Sci..

[17] Zhou S., Chen Z., Dichmont C.M., Ellis N., Haddon M., Punt A.E., Smith A.D.M., Smith D.C., Ye Y. (2016). Catch-Based Methods for Data-Poor Fisheries.

[18] Hilborn R., Walters C.J. (1992). Quantitative Fisheries Stock Assessment: Choice, Dynamics, and Uncertainty.

[19] Ying Y., Chen Y., Lin L., Gao T., Quinn T. (2011). Risks of ignoring fish population spatial structure in fisheries management. Can. J. Fsih. Aquat. Sci..

[20] Cao J., Truesdell S.B., Chen Y. (2014). Impacts of seasonal stock mixing on the assessment of Atlantic cod in the Gulf of Maine. ICES J. Mar. Sci..

[21] Lilly G.R., Parsons D.G., Veitch P.J. (1998). Spatial structure of northern shrimp (*Pandalus borealis*) off Labrador and eastern Newfoundland (Northwest Atlantic). Can. Speci. Publ. Fish. Aquat. Sci..

[22] Ames E.P. (2004). Atlantic cod stock structure in the Gulf of Maine. Fisheries.

[23] Jennings S., Kaiser M., Reynolds J.D. (2009). Marine Fisheries Ecology.

[24] Hart D.R., Cadrin S.X. (2004). Yellowtail flounder (*Limanda ferruginea*) off the northeastern United States: Implications of movement among stocks. Species Conservation and Management: Case Studies.

[25] Michio Y., Tanaka H., Honda S., Nishida H., Nashida K., Hirota Y., Ishida M., Ohshimo S., Miyabe S., Ito H. (2013). Sexual maturation, spawning period and batch fecundity of Japanese sardine (*Sardinops melanostictus*) in the coastal waters of western Japan in 2008–2010. Bull. Jpn. Soc. Fish. Oceanogr..

[26] Suda M., Watanabe C., Akamine T. (2008). Two-species population dynamics model for Japanese sardine *Sardinops melanostictus* and chub mackerel *Scomber japonicus* off the Pacific coast of Japan. Fish. Res..

[27] Yatsu A., Watanabe T., Ishida M., Sugisaki H., Jacobson L.D. (2005). Environmental effects on recruitment and productivity of Japanese sardine *Sardinops melanostictus* and chub mackerel *Scomber japonicus* with recommendations for management. Fish. Oceanogr..

[28] Yang C., Han H.B., Zhang H., Shi Y.C., Su B., Jiang P.W., Xiang D.L., Sun Y.Y., Li Y. (2023). Assessment and management recommendations for the status of Japanese sardine *Sardinops melanostictus* population in the Northwest Pacific. Ecol. Ind..

[29] Okunishi T., Yamanaka Y., Ito S. (2009). A simulation model for Japanese sardine (*Sardinops melanostictus*) migrations in the western North Pacific. Ecol. Model..

[30] Shi Y.C., Kang B., Fan W., Xu L.L., Zhang S.M., Cui X.S., Dai Y. (2023). Spatio-temporal variations in the potential habitat distribution of Pacific sardine (*Sardinops sagax*) in the Northwest Pacific Ocean. Fishes.

[31] NPFC 5th Meeting Report, NPFC-2022-TWG CMSA05-Final Report. Proceedings of the 5th Meeting of the Technical Working Group on Chub Mackerel Stock Assessment.

[32] Helser T.E., Punt A.E., Methot R.D. (2004). A generalized linear mixed model analysis of a multi-vessel fishery resource survey. Fish. Res..

[33] Smith S.J. (1990). Use of statistical models for the estimation of abundance from groundfish trawl survey data. Can. J. Fish. Aquat. Sci..

[34] Thorson J.T. (2015). Spatio-temporal variation in fish condition is not consistently explained by density, temperature, or season for California Current groundfishes. Mar. Ecol. Prog. Ser..

[35] Ono K., Ianelli J.N., McGilliard C.R., Punt A.E. (2018). Integrating data from multiple surveys and accounting for spatio-temporal correlation to index the abundance of juvenile Pacific halibut in Alaska. ICES J. Mar. Sci..

[36] Thorson J.T., Ianelli J.N., Larsen E.A., Ries L., Scheuerell M.D., Szuwalski C., Zipkin E.F. (2016). Joint dynamic species distribution models: A tool for community ordination and spatio-temporal monitoring. Glob. Ecol. Biogeogr..

[37] Pinto C., Travers-Trolet M., Macdonald J.I., Rivot E., Vermard Y. (2019). Combining multiple data sets to unravel the spatiotemporal dynamics of a data-limited fish stock. Can. J. Fish. Aquat. Sci..

[38] Kai M., Thorson J.T., Piner K.R., Maunder M.N. (2017). Predicting the spatio-temporal distributions of pelagic sharks in the western and central North Pacific. Fish. Oceanogr..

[39] Thorson J.T., Adams C.F., Brooks E.N., Eisner L.B., Kimmel D.G., Legault C.M., Rogers L.A., Yasumiishi E.M. (2020). Seasonal and interannual variation in spatio-temporal models for index standardization and phenology studies. ICES J. Mar. Sci..

[40] Thorson J.T. (2019). Guidance for decisions using the Vector Autoregressive Spatio-Temporal (VAST) package in stock, ecosystem, habitat and climate assessments. Fish. Res..

[41] Skaug H., Fournier D. (2006). Automatic approximation of the marginal likelihood in non-Gaussian hierarchical models. Comput. Stat. Data Anal..

[42] Kristensen K., Nielsen A., Berg C.W., Skaug H., Bell B.M. (2016). TMB: Automatic differentiation and Laplace approximation. J. Stat. Softw..

[43] Fournier D.A., Skaug H.J., Ancheta J., Ianelli J., Magnusson A., Maunder M.N., Nielsen A. (2012). AD model builder: Using automatic differentiation for statistical inference of highly parameterized complex nonlinear models. Optim. Methods Softw..

[44] Lindgren F., Rue H. (2015). Bayesian spatial modelling with R-INLA. J. Stat. Softw..

[45] McLeod A.I. (2015). Kendall. https://cran.r-project.org/web/packages/Kendall/Kendall.pdf.

[46] Shi Y.C., Zhang X.M., Yang S.Y., Dai Y., Cui X.S., Wu Y.M., Zhang S.M., Fan W., Han H.B., Zhang H. (2023). Construction of CPUE standardization model and its simulation testing for chub mackerel (*Scomber japonicus*) in the Northwest Pacific Ocean. Ecol. Ind..

[47] Schaefer M.B. (1954). Some aspects of the dynamics of populations important to the management of the commercial marine fisheries. Bull. Inter-Am. Trop. Tuna Comm..

[48] Ji Y., Liu Q., Liao B. (2019). Estimating biological reference points for largehead hairtail (*Trichiurus lepturus*) fishery in the Yellow Sea and Bohai Sea. Acta Oceanol. Sin..

[49] Goethel D.R., Quinn T.J., Cadrin S.X. (2011). Incorporating spatial structure in stock assessment: Movement modeling in marine fish population dynamics. Rev. Fish. Sci..

[50] Zipkin E.F., Ries L., Reeves R., Regetz J., Oberhauser K.S. (2012). Tracking climate impacts on the migratory monarch butterfly. Glob. Chang. Biol..

[51] Sarr O., Kindong R., Tian S.Q. (2021). Knowledge on the Biological and Fisheries Aspects of the Japanese Sardine, *Sardinops melanostictus* (Schlegel, 1846). J. Mar. Sci. Eng..

[52] Ma S.Y., Fu C.H., Li J.C., Sun P., Liu Y., Ye Z.J., Watanabe Y., Tian Y.J. (2022). Non-Stationary effects of multiple drivers on the dynamics of Japanese sardine (*Sardinops melanostictus*, *Clupeidae*). Fish Fish..

[53] Sakuramoto K. (2013). A recruitment-forecasting model for the Pacific stock of the Japanese sardine (*Sardinops melanostictus*) that does not assume density-dependent effects. J. Agric. Sci..

[54] Shelton A.O., Thorson J.T., Ward E.J., Feist B.E. (2014). Spatial semiparametric models improve estimates of species abundance and distribution. Can. J. Fish. Aquat. Sci..

[55] Thorson J.T., Ianelli J.N., Munch S.B., Ono K., Spencer P.D. (2015). Spatial delay difference models for estimating spatiotemporal variation in juvenile production and population abundance. Can. J. Fish. Aquat. Sci..

[56] Thorson J.T., Fonner R., Haltuch M.A., Ono K., Winker H. (2017). Accounting for spatio-temporal variation and fisher targeting when estimating abundance from multispecies fishery data. Can. J. Fish. Aquat. Sci..

[57] Grieve B.D., Hare J.A., Saba V.S. (2017). Projecting the effects of climate change on Calanus finmarchicus distribution within the U.S. Northeast Continental Shelf. Sci. Rep..

[58] Kanamori Y., Takasuka A., Nishijima S., Okamura H. (2019). Climate change shifts the spawning ground northward and extends the spawning period of chub mackerel in the western North Pacific. Mar. Ecol. Prog. Ser..

[59] Harley S.J., Myers R.A., Dunn A. (2001). Is catch-per-unit-effort proportional to abundance?. Can. J. Fish. Aquat. Sci..

[60] Shi Y.C., Han H.B., Tang F.H., Zhang S.M., Fan W., Zhang H., Wu Z.L. (2023). Evaluation Performance of Three Standardization Models to Estimate Catch-per-Unit-Effort: A Case Study on Pacific Sardine (*Sardinops sagax*) in the Northwest Pacific Ocean. Fishes.

[61] Furuichi S., Niino Y., Kamimura Y., Yukami R. (2020). Time-varying relationships between early growth rate and recruitment in Japanese sardine. Fish. Res..

[62] Muko S., Ohshimo S., Kurota H., Yasuda T., Fukuwaka M.A. (2018). Long-term distribution changes in distribution of Japanese sardine in the Sea of Japan during the stock fluctuations. Mar. Ecol. Prog. Ser..

[63] Zhang K. (2022). Fish stock assessment based on abundance index and resilience: A case study of largehead hairtail in Sea of Japan and East China Sea. South China Fish. Sci..

[64] Tameishi H., Shinomiya H., Aoki I., Sugimoto T. (1996). Understanding Japanese sardine migrations using acoustic and other aids. ICES J. Mar. Sci..

[65] Campbell R.A. (2016). A new spatial framework incorporating uncertain stock and fleet dynamics for estimating fish abundance. Fish Fish..

[66] Wang Y., Wang Y., Liang C., Zhang H., Xian W. (2020). Assessment of 12 fish species in the northwest Pacific using the CMSY and BSM methods. Front. Mar. Sci..

[67] Falsone F., Scannella D., Geraci M.L., Gancitano V., Vitale S., Fiorentino F. (2021). How Fishery Collapses: The Case of *Lepidopus caudatus* (Pisces:Trichiuridae) in the Strait of Sicily (Central Mediterranean). Front. Mar. Sci..

[68] Hashimoto M., Nishijima S., Yukami R., Watanabe C., Kamimura Y., Furuichi S., Ichinokawa M., Okamura H. (2019). Spatiotemporal dynamics of the Pacific chub mackerel revealed by standardized abundance indices. Fish. Res..

[69] Maunder M.N., Punt A.E. (2004). Standardizing catch and effort data: A review of recent approaches. Fish. Res..

[70] Ye Y., Dennis D. (2009). How reliable are the abundance indices derived from commercial catch–effort standardization?. Can. J. Fish. Aquat. Sci..

[71] Watanabe Y., Zenitani H., Kimura R. (1995). Population decline of the Japanese sardine *Sardinops melanostictus* owing to recruitment failures. Can. J. Fish. Aquat. Sci..

[72] Noto M., Yasuda I. (1999). Population decline of the Japanese sardine, *Sardinops melanostictus*, in relation to sea surface temperature in the Kuroshio extension. Can. J. Fish. Aquat. Sci..

[73] Matsuyama M., Adachi S., Nagahama Y., Kitajima C., Matsuura M.S. (1991). Annual reproductive cycle of the captive female Japanese sardine (*Sardinops melanostictus*): Relationship to ovarian development and serum levels of gonadal steroid hormones. J. Mar. Biol..

